# An algorithm to parse segment packing in predicted protein contact maps

**DOI:** 10.1186/s13015-016-0080-x

**Published:** 2016-06-18

**Authors:** William R. Taylor

**Affiliations:** Francis Crick Institute, 1 Midland Rd, London, NW1 1AT UK

**Keywords:** Protein structure prediction, Correlated substitution analysis, Contact matrix parsing, Frozen approximation algorithm

## Abstract

**Background:**

The analysis of correlation in alignments generates a matrix of predicted contacts between positions in the structure and while these can arise for many reasons, the simplest explanation is that the pair of residues are in contact in a three-dimensional structure and are affecting each others selection pressure. To analyse these data, A dynamic programming algorithm was developed for parsing secondary structure interactions in predicted contact maps.

**Results:**

The non-local nature of the constraints required an iterated approach (using a “frozen approximation”) but with good starting definitions, a single pass was usually sufficient. The method was shown to be effective when applied to the transmembrane class of protein and error tolerant even when the signal becomes degraded. In the globular class of protein, where the extent of interactions are more limited and more complex, the algorithm still behaved well, classifying most of the important interactions correctly in both a small and a large test case. For the larger protein, this involved examples of the algorithm apportioning parts of a single large secondary structure element between two different interactions.

**Conclusions:**

It is expected that the method will be useful as a pre-processor to coarse-grained modelling methods to extend the range of protein tertiary structure prediction to larger proteins or to data that is currently too ’noisy’ to be used by current residue-based methods.

## Background

In recent years, the huge increase in the number of sequences, combined with a number of new methods for the analysis of correlated mutations (or substitutions) in multiple protein sequence alignments has led to a revival in the interest and ability to predict protein structure from sequence data alone. (See [1] for a review).

Results from the analysis of correlation in alignments take the form of predicted contacts between positions in the structure and while these can arise for many reasons, the simplest explanation is that the pair of residues are in contact in a three-dimensional structure and are affecting each others selection pressure.

The construct of a contact or distance matrix (or plot or map) has been used in the analysis of protein structure for many years and is still often referred to as a Phillips or Ooi plot from its original use in the early 1970s [2, 3]. The data in the plot contains almost all the information required to reconstruct the 3D protein (with the exception of chirality) and as such has been used in many methods that deal with either observed distances [4, 5] or predicted distances [6, 7] and also in the comparison of 3D structures [8, 9]. (See [10] for a review of all these areas).

These early attempts to construct protein 3D structures from predicted distances were hampered by the poor quality and specificity of the predicted distance estimates (which were based largely on just the preference of hydrophobic amino acids to lie closer together than average). With data from correlated mutations, specificity has been greatly improved but in many situations, the quality of the data is too poor to directly construct a model using the distance-geometry methods mentioned above.

**Fig. 1:**
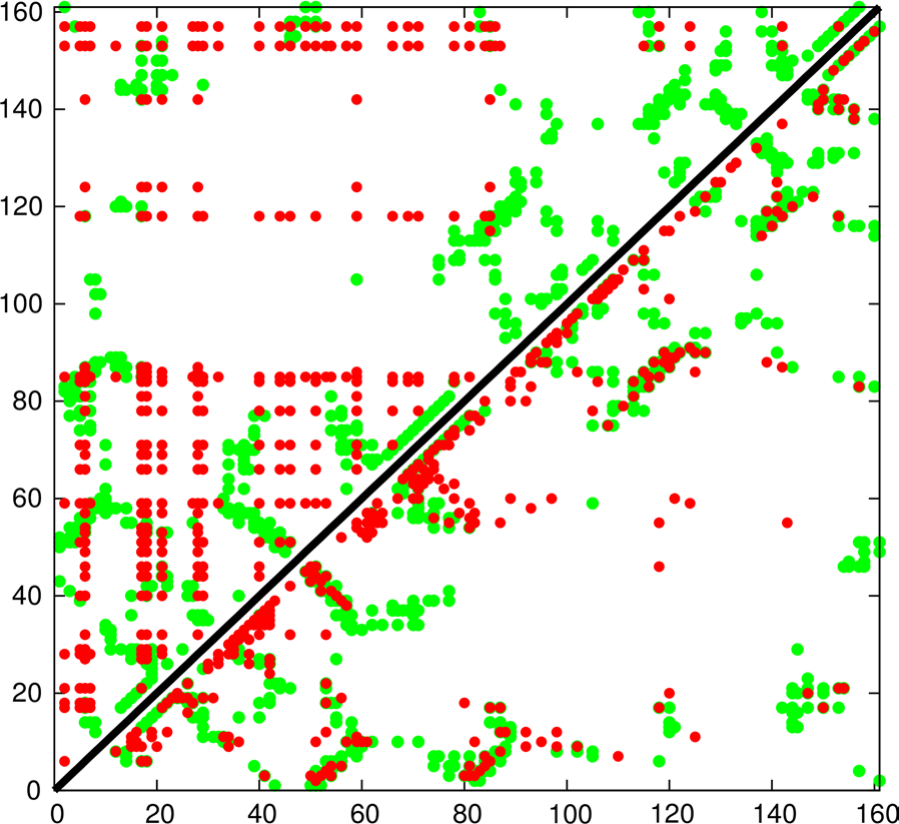
Comparison of predicted contacts for a typical domain sized protein (160 residues). The observed residue contacts are plotted in *green* and as the plot is symmetric the same contacts appear either side of the diagonal. In the *top-left half*, contacts predicted by the generic measure of conserved hydrophobicity are plotted in *red* which produces a characteristic “tartan” pattern. Contacts predicted using the correlated substitution method (PSICOV) are plotted in the *lower-right half*. These predicted contacts exhibit a clear pattern of diagonal and cross-diagonal stripes that are a much better match to the observed packing. Reproduced from Ref. [1], with permission

An approach to improve the success of these methods is to use a more coarse-grained approach and to group the predicted pairwise residue interactions into groups that are linked in a common secondary structure which can then be treated as a rigid body. Early attempts at this tended to focus on pattern-matching combined with combinatorial enumeration [11–15], however, these predicted contacts are generic in nature making it difficult to distinguish packing orientation. For example if two helices, A and B, each have a hydrophobic position at either end, An and Bn (amino ends) and Ac and Bc (carboxy ends) then all pairs (An/Bn, An/Bc, Ac/Bn, Ac/Bc) will appear equal and will, in general, lead to a “tartan” pattern such as that shown in Fig. 1 (top half).

By contrast, the method of correlated mutations predicts residue contacts that are specific and so can in principle distinguish the packing orientation of secondary structure segments. This produces a pattern of contact strips that run parallel to or orthogonal to the diagonal of the plot as shown in Fig. 1 (lower half). This pattern of contact is easily distinguished from the generic “tartan” pattern.

The aim of this work is to automatically identify these interaction patterns (diagonal stripes) in a contact matrix and to assign them as deriving either from the parallel or antiparallel packing of secondary structure elements (SSEs). In addition as the predicted end-points of the SSE are error prone, the definition of their packing can also be used to refine their end-points. The method is thus a general parser for secondary structure packing in protein contact maps.

## Methods

An inter-residue distance or contact map is a square matrix with a size equal to the length of the protein in which each element holds a value for each distance between two residues or, a sometimes binary, assignment where a contact is defined between them. (Fig. 1, green dots).

Given the constraints of a three-dimensional space and in particular, the constraints imposed by a linear polymer chain, such plots are far from random. This is especially so when the underlying structure contains linear elements of substructure, such as the $\alpha$-helix or $\beta$-strand. If these elements are roughly aligned (as is strictly imposed within a $\beta$-sheet), then the contact map exhibits stripes parallel to or orthogonal to the diagonal indicating parallel and antiparallel packing respectively.

### Packing orientation assignment

#### Moments of inertia

Given the submatrix (box) defined by two secondary structure elements in a contact map, The diagonal/orthogonal trend can be quantified by the strength of fit of a regression line in each direction. Neglecting the angle at which the pair of segments pack (which will affect the gradient of the line), if exact parallel and antiparallel packing is assumed, then the fit to a line passing through a point **o**, will be the sum of the perpendicular distances of all points in the box to the diagonal line. For parallel interactions the line will have gradient 1 and −1 for antiparallel. The perpendicular distance, *h*, for each of these cases is simply: $h^2 = (x^2+y^2)/2 \pm xy$, where *x* and *y* are the coordinates of the point from the line which, in the case of a matrix, are the indices of the matrix element relative to **o**. The solution with $+xy$ is for parallel and $-xy$ for antiparallel interactions^1^. The root mean sum of the squared distances to each diagonal over all elements in the box is equivalent to the moment of inertia about an axis. As each element has a strength, this can be treated as a weight giving:

1 $$\begin{aligned} M^2_{pq} = \frac{1}{W} \sum ^a_{i=1} \sum ^b_{j=1} w_{ij}((i-p)^2 +(j-q)^2 \pm (i-p)(j-q)), \end{aligned}$$

where the point {*p*, *q*} lies at the intersection of the diagonal and orthogonal lines and {*i*, *j*} is an element with weight $w_{ij}$ in the surrounding box with dimensions *a* and *b*. *W* is the sum over all $w_{ij}$ in the box and the signs are chosen for parallel/antiparallel as $+/-$ (as for the $\pm xy$ term above), giving two quantities designated $M_P$ and $M_A$, respectively.

As we are interested in a relative score between the lines, this was quantified by the ratio (*R*) of the parallel moment ($M_P$) and the antiparallel moment ($M_A$) as:

2 $$\begin{aligned} R = \left\{ \begin{array}{ll} -(M_P+1)/(M_A+1) +1 &{} \text{ if } M_P>M_A \\ (M_A+1)/(M_P+1) -1 &{} \text{ otherwise } \end{array} \right. . \end{aligned}$$

The moments were modified by adding 1 to avoid extreme values when all points lay close to or on the line and as the ratio is always greater than 1, the value of 1 was subtracted and the terms arranged to give positive values for parallel packing and negative values for antiparallel. (Note that the moment of inertia is large when values lie away from line and small when close.)

The ratio *R* was calculated for the point {*p*, *q*} that lies at the weighted centroid of the box. The sign of *R* then specifies parallel/antiparallel packing and its magnitude indicates the strength of the orientation bias.

#### Central overlap bias

In its current general formulation, the ratio score, *R*, might find a solution where the diagonal line is confined to a corner of the box. Although this may reflect the true state of packing for a staggered pair of secondary structure elements^2^, it would be better avoided in favour of a more central solution, even if this is slightly lower in score. To implement this bias, an additional term was introduced as a Gaussian weight from the centre of the box and applied to the calculation of the centroid position, **c** = {*p*, *q*}. For a box with dimensions *a* and *b*:

3 $$\begin{aligned} \text{ c } = \frac{1}{W'} \sum ^a_{i=1} \sum ^b_{j=1} w_{ij} \exp (-d^2/s^s)\text{m}, \end{aligned}$$

where, **m** is the point at {*i*, *j*} which lies at a distance *d* from the centre of the box ($d^2 = (i-a/2)^2+(j-b/2)^2$), *s* is the Gaussian damping term (*c.f.* standard deviation) and $W'$ is now the sum over all $w_{ij}$ values in the box, similarly weighted:

4 $$\begin{aligned} W' = \sum ^a_{i=1} \sum ^b_{j=1} w_{ij} \exp (-d^2/s^2). \end{aligned}$$

The ’default’ value of *s* was assigned as 10 and the results indicated no reason to change this.

#### Refined diagonal selection

Having established a preferred orientation, the position of the diagonal line was refined by down-weighting out-lying points. While an analytic form may exist for this, the simpler approach was taken to evaluate the weighted moment for each diagonal using Eq. 1 but selecting a point {*p*, *q*} on each diagonal line to be tested, between the limits of {$a-b/4,b/4$} and {*b*/4, 3*b*/4} which excludes two opposing corners of a box having sides of length *a* and *b* (with $a>b$).

The perpendicular distance to the line (*h*) was then weighted by a Gaussian function giving: $h' = h \exp (-h^2/r^2)$, where $h^2 = (i-p)^2 +(j-q)^2 \pm (i-p)(j-q)$ and *r* is a damping term. (*r* was also assigned a default value of 10 which remained unchanged.) By alternately incrementing the values of *p* and *q* across the range defined above, the diagonal line (passing through {*p*, *q*}) was then found that gave a minimum weighted sum of squares of the predicted packing values (*w*). The moment (*M*) of the predicted packing scores about this best line is thus:

5 $$\begin{aligned} M^2 = \frac{1}{W'} \sum ^a_{i=1} \sum ^b_{j=1} w_{ij}{h'}_{ij}^2, \end{aligned}$$

where $h'_{ij}$ is the distance-weighted perpendicular to the best diagonal line and $W'$ is the distance-weighted sum of all scores in the box (from Eq. 4).

### Segment end-point refinement

As mentioned above, the diagonal packing assignment might be improved or extended by altering the end-points of the two secondary structure elements (SSEs) that define the interaction box used in the calculation. This would not be an unreasonable approach as there is generally a substantial error associated with the prediction of SSEs and by optimizing the degree of predicted packing between them, the definition of the elements themselves might even be improved.

This approach is similar to the applications to transmembrane segment prediction [16] and parsing linear 3D structural segments [17] in both of which an optimal solution can be obtained using the dynamic-programming algorithm. However, in these applications, the evaluation score for a single SSE depends only on its own two end points, calculating the sum of the propensity for amino acids to be in a membrane environment and a measure of how long and thin a set of 3D points appear, respectively. (The latter being defined by the moments of inertia of the point-set).

Despite clear similarity to the latter application, the current problem involves, as a minimum two SSEs and, in general, for every end-point that is altered, all interactions involving that element will need to be recalculated. This non-local aspect to the problem is therefore more equivalent to the comparison of structural environments between two protein structures [8] or the problem of “threading” a sequence over a given structure [18]. These more complex problems (which are actually NP-complete [19]) have been solved by a variety of methods ranging through brute-force stochastic approaches (e.g., Monte Carlo, simulated annealing), the “frozen-approximation” algorithm (or FAA, in which each component is optimised in turn while the others are held fixed) [20], to double-dynamic-programming (DPP), which is a two-level dynamic programming algorithm [8]. (See chapter 15 in ref. [21] for an explanation of all these methods).

Although the number of SSEs is generally small in a protein of moderate size, each end can be displaced up and down independently giving a large combinatorial space to be explored so stochastic search methods were avoided. Unfortunately, the DDP algorithm does not map easily to the current problem so it too was not tried. However, given that there are reasonable predicted estimates for the SSE end-points and a deterministic dynamic programming algorithm to find a local solution, the FAA was adopted as the most practical alternative.

#### Iterated frozen approximation algorithm

For a set of given SSE end-points **A**, diagonal packing assignments (*M*) can be found as described above for each pair of SSEs (Eq. 5) and summed to give an overall score ($M_T$) as:

6 $$\begin{aligned} M_T = \sum _{i=1}^N \sum _{j=1,j>i}^N M_{ij}, \end{aligned}$$

where *i* and *j* specify a pair of SSEs with end-points $\mathbf {a}_i =\{a_{i,1},a_{i,2}$}, etc., (with $a_{i,1}$ and $a_{i,2}$ being the start and final sequence positions of SSE *i*), with the full set of *N* end-points, **A** = {**a**$_1,\ldots$**a**$_N$}.

For any individual segment, *k*, its partial score will be:

7 $$\begin{aligned} M_k = \sum _{j=1,j\ne k}^N M_{kj}, \end{aligned}$$

Following the dynamic programming formulation of Taylor [8], as used previously for the definition of linear (3D) segments, a score matrix (**T**) was constructed with the protein sequence (of length *L*) along one axis ($R = {r_1 \ldots r_L}$) and segment size (S = {$s_1 \ldots s_{L/2-1}$}) along the other. At any sequence position $r_i$, segment size is specified as an equal displacement of $\pm s_j$ from $r_i$ giving a segment length of $2s_j+1$ with ends $r_i-s_j$ and $r_i+s_j$. This segment is represented by the element $\mathbf{T}_{ij}$ and the full matrix **T** thus specifies every possible segment size^3^ at every position in the sequence.

**Fig. 2:**
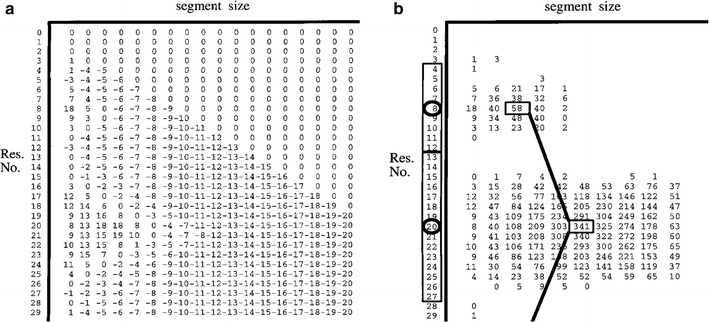
Dynamic programming score matrix construction. In both parts, a score matrix is shown with the protein sequence (Res. No.) running down and segment size running* left* to* right*. Any position thus specifies a unique segment on the sequence. In part ($\mathbf a$) the raw scores $M_k$ from Eq. 7 are entered, minus a penalty against large segments that increases linearly with larger segment size. In part ($\mathbf b$) these scores have been summed over the *triangle* lying *below* each segment position (only positive scores are now shown for clarity) and a dynamic programming algorithm selects the highest sum of scores under the constraint the segments do not overlap. For the two segments illustrated (*joined boxes*), the scores are locally maximal for each segment but if, say, the 40 to the* right* of the* boxed* 58 had been 60 instead, then a larger first segment would have been selected at the small cost of displacing the second segment down one position where it still scores 340, giving a higher total score Reproduced from Ref.[17], with permission

However, not all elements of **T** are valid and segments spanning the sequence termini are excluded, giving a triangular array from $L-2$ segments of length 3 at the base to one segment of length *L* at the apex. Also, segments should not overlap so, given the current SSE end-point assignments (**A**), if position $r_i$ lies in SSE **a**_k_ (*i.e.*, $r_i \ge a_{k1}$ and $r_i \le a_{k2}$), then if $r_i-s_j \le a_{k-1,2}$ or $r_i+s_j \ge a_{k+1,1}$, the segment $\mathbf{T}_{ij}$ overlaps its neighbours and is excluded. Given these constraints, each valid score matrix element ($\mathbf{T}_{ij}$) is assigned the value $M_k$ (Eq. 7) calculated using **A** but with $a_{k,1} = r_i-s_j$ and $a_{k,2} = r_i+s_j$ whenever $r_i$ falls in $\mathbf{a}_k$. Given this score matrix construction, the dynamic programming algorithm finds the combination of segments that gives a maximum value for $M_T$ (Eq. 6; see Fig. 2).

Although the end-points of each SSE are re-calculated simultaneously by the dynamic programming algorithm, this has been done for each element using the fixed endpoints of all the others which have been treated as a “frozen approximation” of their optimal values. If these initial end-points were reasonable, then a single pass of the algorithm would probably suffice, however the new definitions may allow further improvement so the algorithm should be iterated.

If **A**$_0$ is the starting set of ends, say, from a secondary structure prediction algorithm, then applying the dynamic-programming algorithm will generate a refined set ($\mathbf{A}_1$). If the process is iterated, then in general, $\mathbf{A}_t \rightarrow \mathbf{A}_{t+1}$ and when $\mathbf{A}_t = \mathbf{A}_{t-1}$, the process has converged.

#### Structure modified diagonal

Predicted contacts tend to be “noisy”, however, when large numbers of sequences can be gathered for analysis, there is the possibility to infer a packing angle from the contact pattern. An approach using a general regression fit to the data would be possible but, from a survey of the data in the “Results” section, is unlikely to be generally applicable. However it is obvious from the contact plots of the globular proteins, that many lines fall far from the expected 1:1 diagonal gradient.

This more dominant effect on the gradient of the optimal diagonal derives from variations in the helical periodicity (or rise per residue along the axis) of different secondary structure types. Fortunately, in proteins, there are only two dominant secondary types: the $\alpha$-helix, with a period of 1.5 Å/residue and the $\beta$-strand, with a more variable period spanning the range 2.5–3.5 Å/residue. (See ref. [17]; Fig. 8 for a plot of these data from known structures). A good approximation is therefore to take the ratio 1:2 for the relative size of the $\alpha$:$\beta$ periodicities.

If two SSEs of the same type with the same length pack with aligned axes, then their interaction stripe in the contact plot will lie on a 1:1 diagonal line. However, if one of the elements has double the number of residues over the same length, then the slope of the diagonal packing stripe will run between the corners of a rectangle with sides in the ratio 1:2 and this factor will need to be accommodated in the calculation of the best fit.

To do this, the simple method of calculating the moment of inertia about the 1:1 diagonal was retained but when one SSE was an $\alpha$-helix and the other was a $\beta$-strand, then the interaction box was “padded” with alternating rows (or columns) of zeros along the dimension corresponding to the $\beta$-strand, allowing all aspects of the calculation to remain unchanged. These “dummy” rows were, of course, removed when the best fit line was marked on the full contact plot and were unnecessary when analysing the interaction of two $\beta$-strands.

### Sequence analysis

Sequences were extracted from the non-redundant NCBI sequence databank using the jackHmmer program [22] and reduced to a small non-redundant selection for sequence analysis [23] which included secondary structure prediction using PsiPred [24] at: http://bioinf.cs.ucl.ac.uk/MetaPSICOV/.

#### Coevolution analysis

Each of the co-evolution analysis methods described below perform their own sequence search using either jackHmmer or a similar program. In each server we accepted the default search parameters. (These methods do not use the reduced alignment referred to above).

**PSICOV**: Sequences were submitted to the PSICOV server [25] at: http://bioinf.cs.ucl.ac.uk/MetaPSICOV/.

**GREMLIN**: Sequences were submitted to the Gremlin server [26] at: http://gremlin.bakerlab.org/submit.php.

#### TM-helix prediction

TM helices were predicted as a consensus of the methods calculated by the TOPCONS server [27] at: http://topcons.cbr.su.se/pred/.

## Results

### Transmembrane proteins

**Fig. 3:**
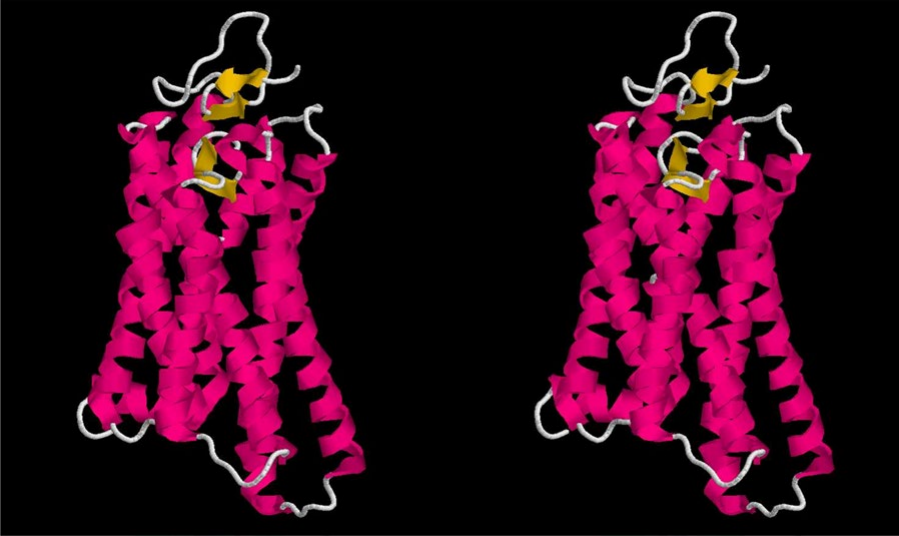
Rhodopsin structure. The eukaryotic rhodopsin structure (PDB:1gzmA) is shown as a stereo backbone cartoon as rendered by the program RASMOL [31] with secondary structures coloured as: *magenta* $\alpha$-helix and *yellow* $\beta$-strands. The membrane runs horizontally through the *middle* of the molecule at right angles to the page. The seven transmembrane helices run back and forth through the membrane

**Fig. 4:**
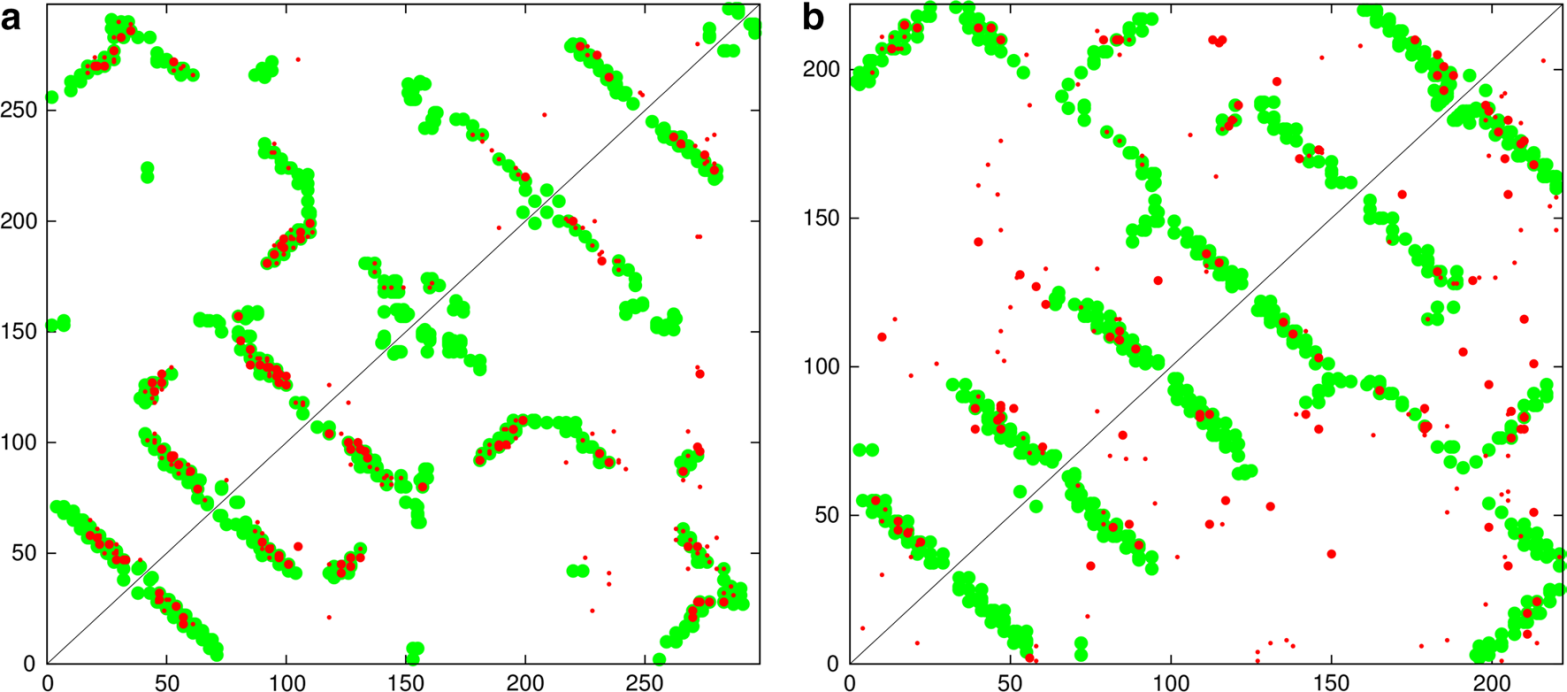
Rhodopsin contact maps. $\mathbf a$ The predicted contacts (*red*) are compared with the observed (*green*) from the structure of rhodopsin (PDB code:1gzm). *Top left* are the top scoring 180 contacts predicted by the GREMLIN method and *bottom-right*, from the PSICOV program. $\mathbf b$ A similar *plot* for the bacteriorhodopsin protein (PDB code: 2brd). The *top* 50 highest scoring contact predictions have a slightly *larger dot* and both sets omit sequence separations under five

An integral transmembrane (TM) protein was selected for initial analysis as the helices in this class of protein tend to be longer than those found in a globular protein of a similar size, giving a clearer signal for testing and development purposes. The well characterised family of rhodopsins was chosen (Fig. 3), using both the large family of eukaryotic GPCR proteins that include various opsins, olfactory proteins and neurotransmitters and the smaller family of bacteriorhodopsins which act as a proton pump in some halophylic species. The first family has almost 200 thousand sequences which generates a relatively clear signal compared to a few thousand for the bacterial family which have a correspondingly greater degree of “noise” in their predicted contacts (Fig. 4).

#### Fixed segment endpoints

**Fig. 5:**
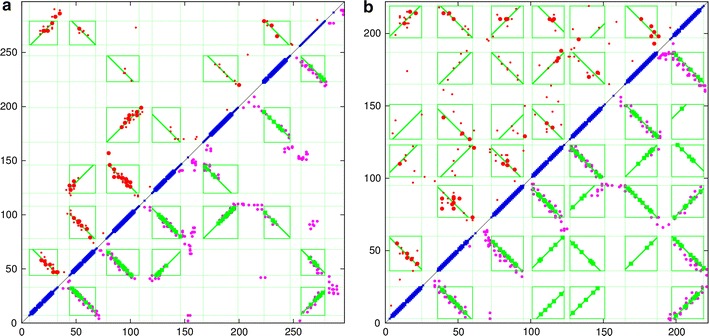
Rhodopsin parsed contact maps (fixed ends). The TM segments predicted by TOPCONS are plotted in *blue* along the diagonal (with a larger mark where all methods agree). Each pair of segments defines an interaction* box *(*green*) and the predicted contacts (*red dots*, *top left*) that fall within a* box *are analysed to determine a preferred packing orientation (*green lines*). In the *lower right half* of the plot, the lines are marked at the points corresponding to the orthogonal projection of the predicted points onto the line. For comparison, the contacts observed on the known structure are marked with *magenta dots*. **a** Data for the eukaryotic rhodopsin family (PDB code: 1gzm) and **b** a similar* plot* for the bacteriorhodopsin protein (PDB code: 2brd)

The basic algorithm was tested initially without iteration using the TM segment endpoints predicted by TOPCONS and the results for both rhodopsin families using the GREMLIN contacts are shown in Fig. 5. (See the “Methods” section for details of the prediction methods).

For the eukaryotic family, it is clear that the algorithm has made the obvious choice of packing line assignment in every box. Given the clarity of the signal with this family, any mis-assignment would have been easily identified (Fig. 5a).

For the bacterial family (Fig. 5b), most of the assignments appear to be visually correct and consistent with the expected up/down packing expected as the helical segments cross the membrane back and forth. Only one assignments is inconsistent with this simple topology: which is between TM segments 1 and 4. (TM segments are the blue diagonal bars in the plots numbered from 1 at the bottom left to 7, top right) (Fig. 5b). However, this pair has a weak signal which would be difficult to assign visually.

**Fig. 6:**
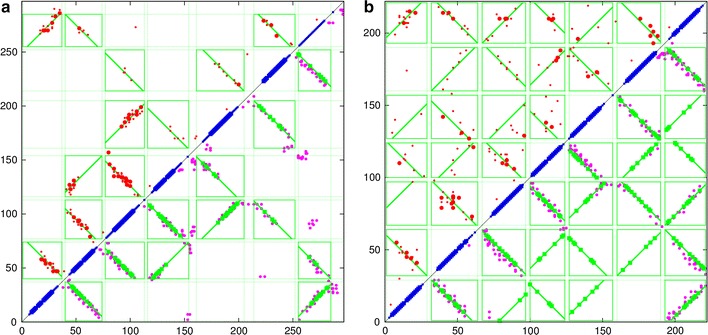
Rhodopsin parsed contact maps (free ends). The data plotted in Fig. 5 was re-analysed with an iterated refinement of the segment end-points. For the eukaryotic protein ($\mathbf a$), little changes except that the first and last segments now include more residue pairs in their interaction* box* (*top left*). For the bacterial family ($\mathbf b$), an incorrect orientation has been corrected between segments 1 and 4 (*middle left edge*)

#### Free segment endpoints

Although most of the fixed TM endpoints corresponded well with the extent of the predicted packing interactions, a clear example of a truncated interaction can be seen in the top-left corner involving the packing of TM segments 1 and 7. To see if this could be corrected, the program was then run with the activation of the full iterated dynamic programming algorithm, taking five cycles to allow progressive refinement of the “frozen” endpoint approximations provided by the TOPCONS prediction.

The results from this run extended most helix endpoints to varying degrees and incorporated the “lost” pairs between segments 1 and 7 in the eukaryotic protein (Fig. 6a). Only one of the top 50 interactions (bigger red dots) now lies outside an interaction box. For the bacterial protein (Fig. 6b), there were less obvious changes due to the shorter loop regions in this proteins, however, the only segment pair (1 and 4) to be mis-assigned previously in the alternating criss-cross packing pattern had managed to pick-up an additional residue pair and fell into a consistent orientation.

#### Additional observations

Interestingly, from an algorithmic viewpoint, the refined end-points attained their final assignments after just one pass of the dynamic programming algorithm. This is perhaps not unexpected as the predicted starting definitions were reasonable accurate. (Discussed further below).

From a structural viewpoint, it is worth noting that the extension of these segments does not imply that there is a larger portion of the TM-helices in the membrane as it is quite common for TM-helices to continue unbroken out of the membrane and still continue to pack together.

The attainment of a perfect criss-cross packing pattern can generally be taken as a sign that the interactions are meaningful and that there are no helices that have been completely missed by the prediction. However, this cannot be guaranteed as exceptions exist where a “re-entrant” segment can dip in then out of the membrane on the same side or simply run along the membrane surface.

In the better quality data for the eukaryotic protein, there was no indication that anything other than a purely parallel/antiparallel packing model was required. From this it might be assumed that discriminating deviations from aligned packing is beyond the resolution of the predicted contacts, given that there is an apparent twist between pairs of helices in the structure (Fig. 3). However, pairs of helices often twist together as if forming a short segment of a coiled-coil (or double helix). This means that contacts between them maintain the same spacing which will still be seen as an exact diagonal line.

### Globular proteins

The contact maps of globular proteins, whether predicted or derived from a known structure, tend to be more complex that those for helical transmembrane proteins. As the name implies, they are more globular in nature which means that for the same sized protein, fewer residues are required to cross from one side of the protein to the other and, without the constraint of the membrane, chain segments are not constrained to make a complete transversal. For the same reason, SSEs are also less restricted in their packing angles which leads to fewer simple patterns to recognise.

In addition, the two types of secondary structure can co-exist in the same protein and although this does not, in principle, affect the analysis of their packing, $\beta$-strands are roughly double the length of an $\alpha$-helix with the same number of residues. This means that for a $\beta$-strand exactly aligned with an $\alpha$-helix of the same spatial length, their interaction box will have a rectangular shape with interacting pairs falling along a corner-to-corner diagonal. As described in the “Methods” section, this correction was applied to the calculation of the best-fit lines for pairs of mixed type SSE interactions.

**Fig. 7:**
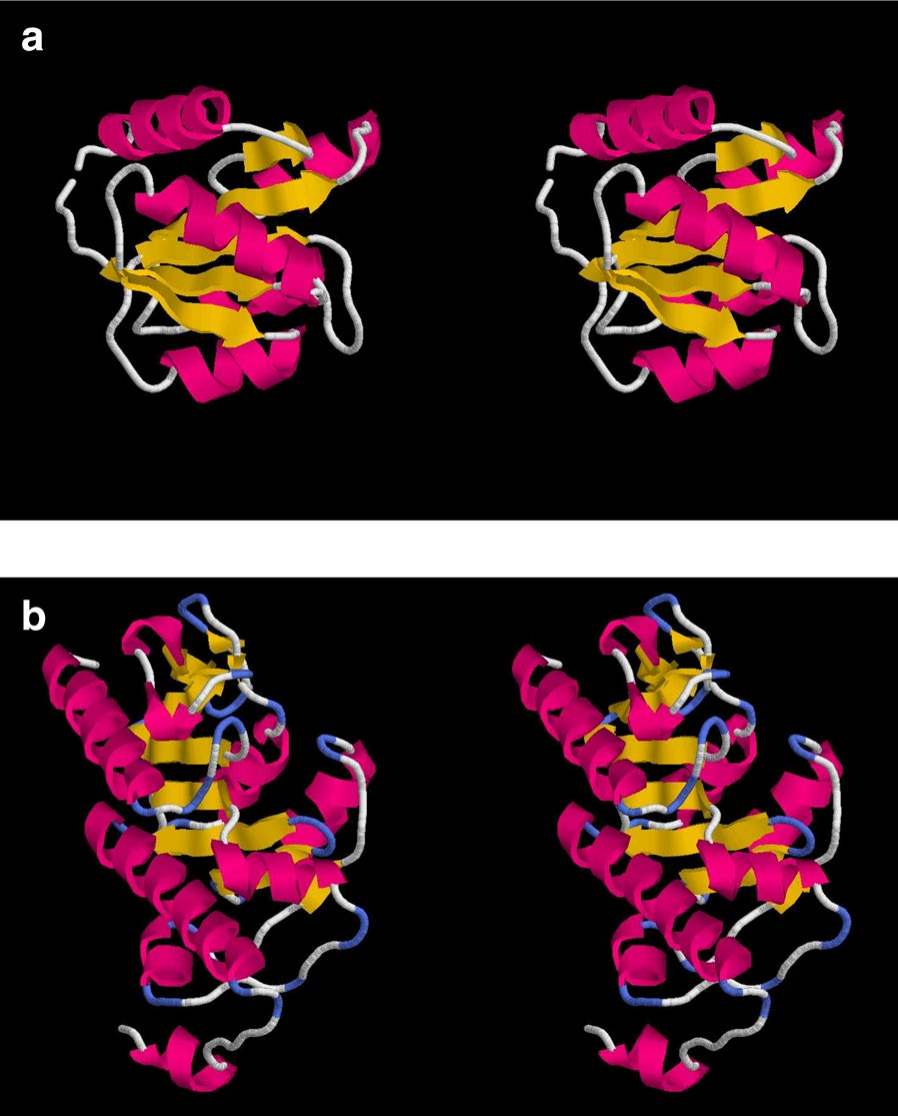
B/A test proteins. The two proteins used as test data for the methods are depicted as in Fig. 3. $\mathbf a$ The chemotaxis-Y protein (PDB:3chyA, len=128) and $\mathbf b$ l-fuculose-1-phosphate aldolase (PDB:2opiA, len=202). In both views, the termini lie on the *left*

With this complication in mind, two proteins of the $\alpha$/$\beta$-class were selected as test data, both have been used previously for testing structure prediction [28]. The first is the small bacterial chemotaxis-Y protein (PDB:3chyA, 128 residues) which has a very large family and consequently good contact and secondary structure predictions and consists of a central $\beta$-sheet packed above and below with $\alpha$-helices. The second protein (PDB:2opiA, 202 res.) is a larger variant of the same type but almost double in length with a much more complex fold and longer loop regions between SSEs (Fig. 7). For both of these, the contacts were predicted by GREMLIN and the secondary structures by PSIPRED. (See the “Methods” section for details of the prediction methods).

#### Choice of “gap”-penalty

The basic dynamic programming algorithm used by the current method has the equivalent of a gap-penalty in sequence alignment to penalise large segments (see Fig. 2 and legend for details). In the application to the rhodopsin data, the segments were sufficiently well defined that a “token” gap-penalty of 1 was used (relative to segment scores over 100). However, from initial trials with the globular class of protein this was found to be unsatisfactory.

Although the algorithm is forbidden to include two SSEs in one segment, this is only defined in terms of the segments from the previous iteration (not the starting prediction) and if one of these is deleted then the neighbouring segments can claim its space on the next iteration. If a starting SSE is an erroneous prediction then this is desirable behaviour, however, it also allows potentially large segments to “swallow” their weaker neighbours even when they are correct. A way to prevent this is to increase the gap-penalty.

For the two proteins considered in this section, the gap-penalty was increased from 1 in unit steps until every predicted segment retained an interaction box. This occurred at a value of 4 for both proteins and further increases over this produced no apparent change, even at high values. A ’default’ value of 5 was used in the results below.

#### Chemotaxis-Y protein

**Fig. 8:**
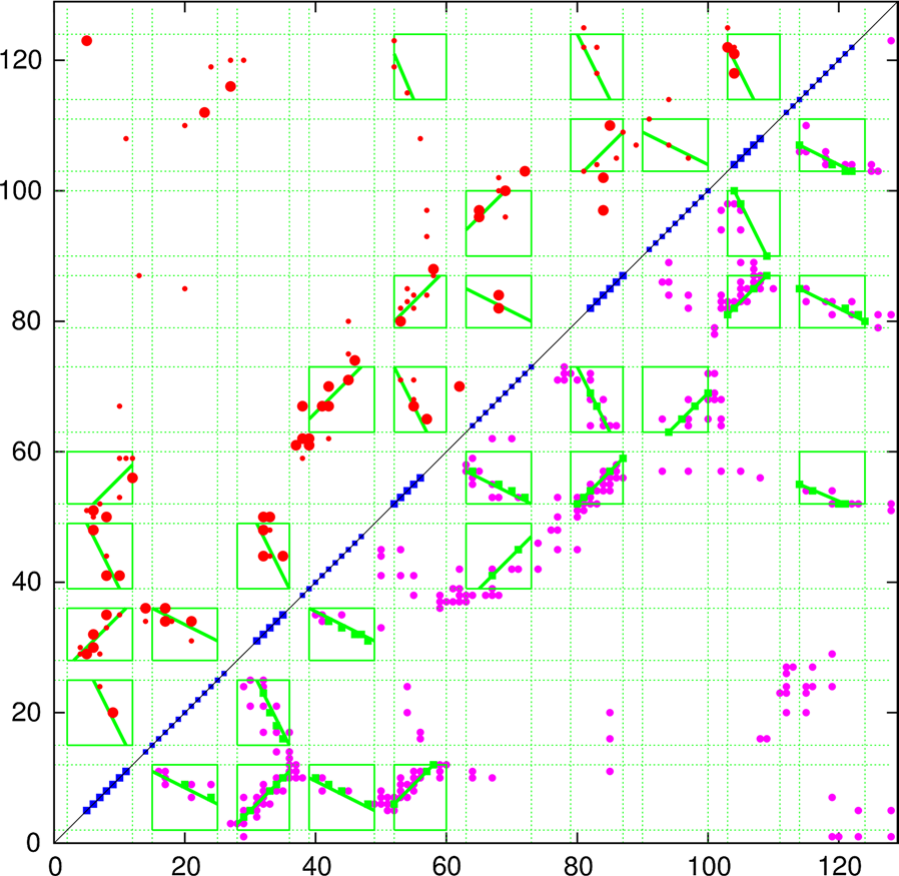
Parsed contact map for 3chyA. The contacts predicted by GREMLIN (*red*) are plotted for the small $\beta$/$\alpha$ protein 3chyA using the same conventions as Fig. 6a, with the exception that the *blue diagonal marks* are now smaller for an $\alpha$-helix and larger for a $\beta$-strand

The parsed contact for the smaller protein PDB:3chyA, is shown in Fig. 8 (using the same representation as the rhodopsin plots in Fig. 6a). The data exhibits some clear packing interaction “stripes” which can be seen to be a good reflection (literally) of the observed packing. However, a few interactions are not predicted including two pairs of adjacent $\beta$-strands and helices: $\alpha$2–$\beta$3 and $\beta$4–$\alpha$4. The latter contains a single predicted contact and as this cannot show any parallel/anti-parallel preference it scores zero and is not reported by the program. The interaction between the terminal helices was also missed, despite some nearby contacts.

Of algorithmic interest is the treatment of the numerous interactions between $\beta$-strands and $\alpha$-helices, where it can clearly be seen that the program has applied the appropriate correction to their diagonal gradients. (e.g., between $\beta$1−$\alpha$1 and $\alpha$1−$\beta$2, lower left in Fig. 8).

#### L-Fuculose-1-phosphate aldolase

Compared to rhodopsin and the smaller chemotaxis-Y protein, the structure of L-fuculose-1-phosphate aldolase (2opiA) contains a number of more complex interactions, including long helices that interact with more than one other SSE along their length and adjacent elements that continue along the same direction. In general, the interactions between SSE were well predicted and even using an older data set, were sufficient to allow the correct fold of the protein to be predicted [28].

In the current data, a few interactions were missed by the parsing algorithm: one was between a minor helix ($\alpha$3) and a hairpin loop ($\beta$7−$\beta$8) and between the following small helix, $\alpha$4, and the start of $\alpha$6. As the algorithm does not parse loop interaction, the former is expected and for the latter, only a single weak contact was predicted. In the same region, an $\alpha$/$\beta$ interaction was also missed between $\alpha$5 and the following $\beta$6, also because of a weak prediction.

**Fig. 9:**
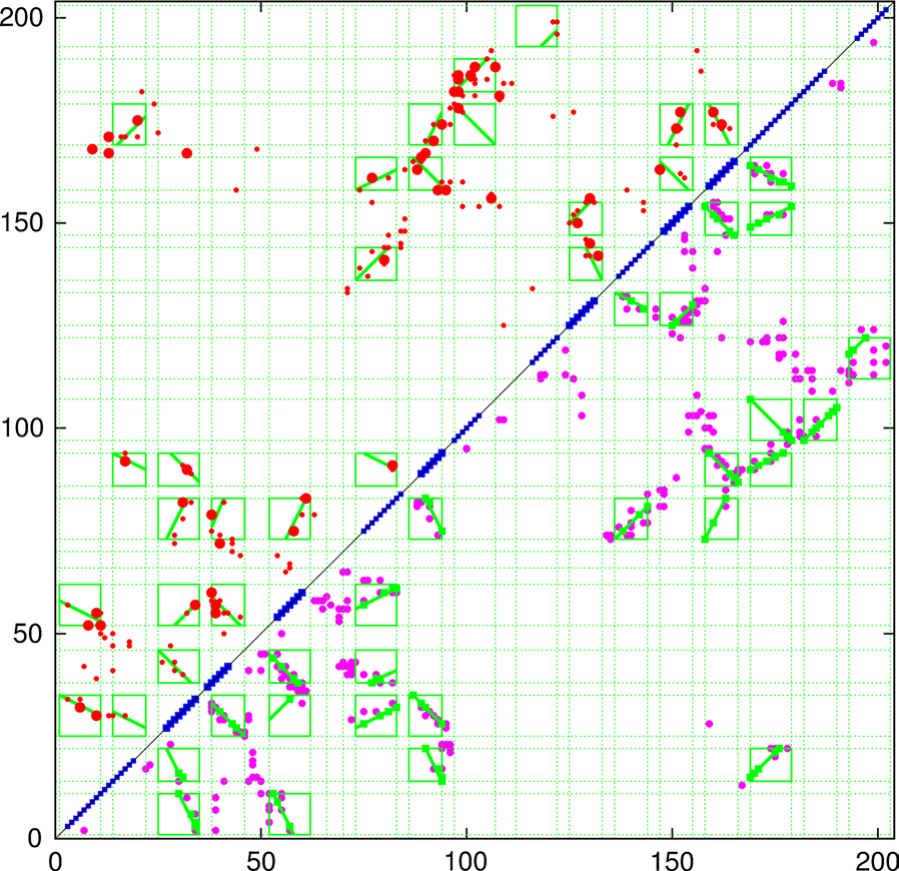
Parsed contact map for 2opiA. The contacts predicted by GREMLIN (*red*) are plotted for the larger $\beta$/$\alpha$ protein 2opiA using the same conventions as Fig. 8

From an algorithmic viewpoint, it was interesting to note that a parallel interaction was correctly identified between two adjacent SSEs ($\beta$3−$\alpha$2) which have a long loop between then that allows such packing. (Showing that the criss-cross packing pattern should not be universally expected).

Some unexpected behaviour was observed for the two long helices which were both parsed into two separate interactions. Although the algorithm is forbidden to include two distinct SSEs in the same interaction box, there is no constraint to prevent it breaking elements into sub-parts.

One of these interactions, involving the first helix, had an interaction box involving its amino (N) terminal part packing with the first $\beta$-strand and a carboxy (C) terminal part (including some of the following loop) allocated to an interaction with the longer helix near the end of the sequence. (see Fig. 9, left edge).

**Fig. 10:**
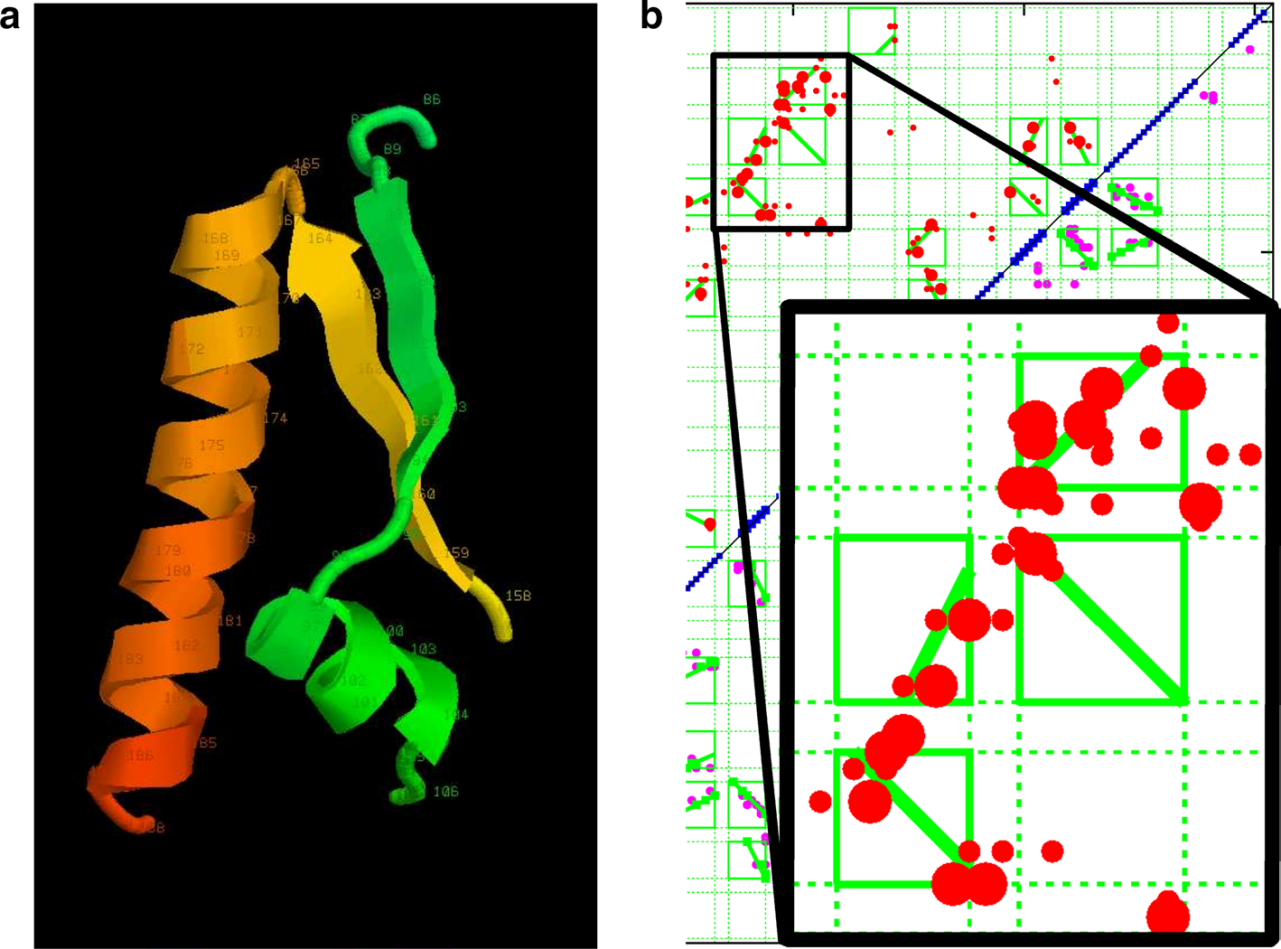
Complex packing parsing. A submatrix from Fig. 9 is examined in detail along with the corresponding structures ($\mathbf a$). The submatrix is expanded as an *inset* in $\mathbf b$, showing its relationship to Fig. 9 in the background. The structure fragments are rendered as before but coloured *green* (residues 86–106) and *yellow*/*orange* (residues 159–188)

The second occurrence involved a complex set of packing between $$\beta $$5−$$\alpha $$3 and $$\beta $$7−$$\alpha $$6, which for clarity has been enlarged in Fig. 10b along with the associated structures (Fig. 10a). In this interaction, it can be seen that the first $$\beta $$$$\alpha $$ pair (green) is extended and $$\beta $$5 interacts with the the N-terminal portion of the helix (orange) whereas $$\alpha $$3 has the C-terminus. (Fig. 10b, top right interaction box in the enlarged inset). The preceding strand ($$\beta $$7) is also correctly identified as an forming an antiparallel interaction with its neighbour ($$\beta $$5, yellow).

## Discussion

### Summary of the results

The simple algorithm developed above for parsing secondary structure interactions in predicted contact maps has been shown to be effective in the transmembrane class of protein and error tolerant even when the signal becomes degraded.

In the globular class of protein, where the extent of interactions are more limited and more complex, the algorithm still behaved well, classifying most of the important interactions correctly in both a small and a large test case.

For the larger protein, this involved examples of the algorithm apportioning parts of a single secondary structure between two different interactions.

### Algorithmic evaluation

#### Need to iterate?

The method was initially designed to use an iterated frozen approximation algorithm (FAA), simply on the theoretical grounds that the starting end-points should not be optimal. In the application of the algorithm, despite the end-points being redefined after the first pass of the dynamic programming algorithm, they were not seen to move again except when using a low gap-penalty with the globular proteins that allowed large segments to ’steal’ their neighbours space. With hind-sight, this may be the expected behaviour as the dynamic programming algorithm will make an optimal segment assignment on the first pass almost certainly by expanding each segment to include more interactions. There will therefore be no scope for further expansion unless a segment is removed. As there can exist situations were weak predictions may be better removed, the iterated algorithm was retained but with just three cycles.

### Undefined starting ends

A drawback of the frozen approximation is that initial values need to be provided and these can sometimes only be obtained from unreliable predictions, especially when there is a paucity of sequence data. The current algorithm has a degree of robustness to errors in the starting definitions as we have seen that it can divide long SSEs into multiple interaction regions or, as discussed above, delete weak SSEs. Two methods have recently been developed to recognise patterns in predicted contact maps [29, 30] that could provide more reliable initial data and a combination of these with the current method might be beneficial.

Asumming, even with improved starting points, that some error might still remain, random end-points could be assigned and refined using a stochastic optimisation process (the Genetic Algorithm would be an attractive possibility). An alternative might be to use a recursive divide-and-conquer algorithm. Choosing any element in the score matrix divides the sequence into three pieces: the segment itself and the regions above and below, one of which may have zero length (but not both or there is nothing to pack). Similarly, points can then be selected in these flanking regions, again dividing them each into three pieces, and so on until no unassigned parts of the sequence remain. At this stage, the value of the original starting element can be evaluated and the algorithm back-tracked. If a reasonably large limit can be imposed on the smallest segment, the approach might be practical.

However, the most effective approach would probably be to make use of variation in the biological data found in the multiple sequences (of which there must be many if the predicted contacts are at all useful). Variations in the secondary structure predictions, or random perturbations to them, could all be used to generate initial end-points which could also be combined with variation of the ’gap’-penalty value to allow segment deletion to occur. As the basic algorithm is very fast, taking a fraction of a second to execute, many trials could be made and a selection of the best retained.

### Conclusions

The method described in this work will provide a useful pre-processing analysis of predicted contact data that can then be used in the subsequent prediction of a 3D structure using coarse grained methods. It is hoped that this strategy will help extend the range of protein tertiary structure prediction to larger proteins or to data that is currently too ’noisy’ to be used by current residue-based methods.

## Footnotes

1 In a contact matrix, a diagonal line, **d** and an orthogonal line **d**′ pass through the element (point) **o**. If a point (element) **p** has a displacement *x*, *y* from **o**, then if **d** has a displacement 1,1 from **o** and **d**′ has a displacement −1,1 from **o**, the perpendicular distance from **p** to **d**′ is: $(x+y)/\surd 2$ (from the dot product **p** · **d**.), giving the squared distance as $(x^2+y^2)/2+xy$. Similarly for **d**.

2 This situation is more likely to indicate that the box is in the wrong place and a more general approach to this problem will be described below based on refining the box definition.

3 These will only be the segments of odd lengths. The extra computational complexity of dealing with both odd and even lengths was considered unjustified given the large degree of uncertainty in the data.
